# Stronger together; evaluating consumers and researchers working in partnership

**DOI:** 10.1186/s40900-025-00716-0

**Published:** 2025-05-01

**Authors:** Grace Gard, Joanna Oakley, Kelsey Serena, Michael Harold, Katya Gray, Helen Anderson, Judi Byrne, Jo Cockwill, Jim Cormack, Graeme Down, George Kiossoglou, Peter Gibbs

**Affiliations:** 1 Personalised Oncology Division, WEHI, Melbourne, Australia; 2 Medical Oncology, Western Health, Melbourne, Australia; 3 WEHI Consumer Program, Melbourne, Australia

**Keywords:** Patient and public involvement, Consumer engagement, Evaluation, Co-design, Cancer research

## Abstract

**Background:**

Consumer engagement in medical research should be early, ongoing, and meaningful, to improve patient-centred research design and outcomes. Evaluating the impact of consumer input builds the evidence base needed to sustain and improve ongoing involvement efforts. Multiple evaluation tools are available. Here we explore the landscape of evaluation tools and apply the Australian Clinical Trials Alliance (ACTA) tool to evaluate the impact and process of consumer involvement in a recently completed project.

**Methods:**

The ACTA evaluation tool was administered to all consumers and research team members in the “RECAP” study. The RECAP study is co-designed as a single-arm trial that implemented registry-generated personalised care plans for patients with newly diagnosed rectal cancer. The ACTA evaluation tool responses underwent thematic analysis, and the research and consumer group assessed the utility of the tool.

**Results:**

The ACTA evaluation tool was completed by the entire team, including six consumers and four researchers. Thematic analysis highlighted inclusivity, workload, communication and remuneration as key considerations in the process of consumer involvement. Consumer impact was measured in terms of seven domains of critical research outcomes. Consumer involvement in the RECAP project was identified to be meaningful, respectful, inclusive, and impactful. The evaluation tool was considered appropriate by all respondents, who identified the ease of use, low burden and ability to address both the consumer impact and experience as positive factors. Identified areas for development included the repetitiveness and lack of specify of some questions, and the uncertainty about the desired length of response.

**Conclusion:**

The ACTA evaluation tool was successfully administered to a team of consumers and researchers engaged in a recently completed trial with positive assessment of tool utility.

## Abstract (plain language summary)

Consumer engagement in medical research should be early, ongoing, and meaningful, to improve research design and outcomes. Evaluating the impact of consumer input builds the evidence base needed to sustain and improve ongoing involvement efforts. Multiple evaluation tools are available. Here we explore the landscape of evaluation tools and apply the Australian Clinical Trials Alliance (ACTA) tool to evaluate the impact and process of consumer involvement in a recently completed project. The ACTA evaluation questionnaire was completed by all consumers and research team members in the “RECAP” study. The RECAP study is a co-designed trial that implemented registry-generated personalised care plans for patients with newly diagnosed rectal cancer. The ACTA evaluation tool responses were analysed, and the research and consumer group assessed the usefulness of the tool. Analysis highlighted inclusivity, workload, communication and remuneration as key considerations in the process of consumer involvement for this team of four researchers and six consumers. Consumer impact was measured in terms of pre-identified critical research outcomes. Consumer involvement in the RECAP project was identified to be meaningful, respectful, inclusive, and impactful. The evaluation tool was considered appropriate by all team members, who identified the ease of use, low burden and ability to address both the consumer impact and experience as positive factors. Identified areas for development included the repetitiveness and lack of specificity of some questions, and the uncertainty about the desired length of response.

The involvement of consumers (a term encompassing patients and carers) in medical research should not be a tokenistic, tick-the-box exercise to please external parties. Patients and carers can bring valuable lived experiences and perspectives to medical research. Their meaningful engagement in research can drive patient-centred design, conduct, and outcomes. Consumer involvement should be early, ongoing, and evaluated, to the benefit of all involved.

While the medical research community is increasingly acknowledging that consumer involvement in research is essential for optimal research quality and integrity, there are real challenges and complexities in ensuring that engaging consumers meets best-practice standards [1]. Conscious and considered evaluation and reporting of consumer engagement is the key to maintaining transparent consumer involvement, and facilitating a necessary shift in the mindset and practice of the research community.

Effective evaluation of consumer involvement in medical research is at once urgent, essential, and challenging. Anecdotal evidence of the impact (and, ultimately, the benefit) of consumer involvement in research is rich and compelling, and there is emerging long-term quantitative data to support the involvement of consumers in patient-centred projects such as clinical trials [1]. Evaluation that can be undertaken as a routine part of consumer involvement in individual research projects is the missing component in building an evidence base. This evaluation must usefully inform research teams about their own consumer engagement process and should also provide evidence for funding bodies regarding the impact and cost of meaningful consumer engagement.

Developing standardised evaluation tools for measuring the impact of consumer involvement in medical research remains difficult [2, 3]. The question of measuring process and impact remains pertinent, raising issues of why (what for? ), how, when, and for/from whom research teams should attempt to capture this information, and, whether this information can be captured by a standardised tool at all [4]. Additionally, a case can be made for short-form evaluation tools, such as reporting checklists [5], and for more in-depth, reflective tools, capturing data through interviews or written responses. These evaluations attempt to capture a holistic picture of the impact of consumer involvement on the research team and the specific research project [4].

Our established co-design team, comprising of a clinician researcher, project staff, and consumers with lived experience of cancer, undertook an evaluation of the consumer engagement processes and impact in a recent research project that had focused on producing personalised care plans for patients newly diagnosed with rectal cancer. This analysis took place at the end of the project, which had spanned three years from initiation to completion of evaluations. Our co-design methodology has been one of collaborative, active, and inclusive engagement, with a focus on equal power distribution and appreciating diverse perspectives. Here, we present our evaluation method, findings, and reflections on the evaluation process to contribute a complete picture of an evaluation of/for a collaborative research team.

## The RECAP project

Utilising the existing Walter and Eliza Hall Institute- Colorectal Cancer (WEHI-CRC) database [6] the RECAP project involved generating a personalised care plan for patients to improve understanding of a rectal cancer diagnosis and planned management, (HREC approval 23/WH/91656). This personalised care plan was co-designed by our team of oncologists, project staff, and consumers to provide patients `with important, accessible information about their diagnosis, planned treatment, and follow-up schedule. The patient specific information contained in the personalised care plan was populated from data captured in an existing clinical cancer registry, where patient information is routinely updated in real time. The care plan template includes a patient’s personalised staging, planned treatment and follow up information, and is a useful patient document created with minimal administrative burden on medical and support staff. The care plan was also securely provided to a patient’s general practitioner (GP), ensuring that the entire cancer care team has the appropriate information to support the patient through their cancer journey. This novel approach to patient education, personalised for each patient and sustainably generated from existing resources, aimed to improve a patient’s knowledge about their cancer diagnosis, thereby reducing anxiety and improving treatment compliance. The personalised care plan was delivered to patients at Western Health (Melbourne, Australia) between August 2023 and June 2024, and through formal evaluation was found to be well-received by patients, oncology clinicians, and GPs, with all groups reporting that the personalised care plan added value and was feasible to integrate into routine practice.

Consumer involvement throughout RECAP has been well-planned and meaningful, and has contributed significantly to the success of the project. Consumers were involved from the project design stage and collaborated on the design of the personalised care plan, patient information sheet, evaluation surveys, and semi-structured interview questions. They also contributed to several publications [7, 8], oral presentations, social media promotion and posters presented at national conferences. Consumer engagement involved four to six meetings per year for the lifetime of the project (2022–2024), and consistent bi-directional email communication. The meetings were predominantly virtual, held during working hours and kept to a maximum one hour in duration. Two more informal, in person meetings occurred towards the end of 2022 and 2023 to facilitate team bonding and collegiality. The meetings were chaired by the same clinician throughout the project, to offer continuity and with the aim of adhering to the agenda and keeping meetings to time, while allowing feedback from all participants. Agendas were sent to the team prior to all meetings to allow consumers ample time to do any preparation. Meeting minutes were sent promptly after each meeting following to allow for consolidation and any email queries between meetings. Throughout the project cycle, consumers were encouraged to email the team leader or any members of the group if they had any queries or required any support with different aspects of the project. This project was funded by the Victorian Comprehensive Cancer Centre (VCCC Alliance), and all consumer involvement in this project has been remunerated according to the VCCC Alliance remuneration policy. Ethics approval was obtained from Western Health low risk ethics panel. The team of researchers and consumers undertook an in-depth evaluation of the co-design process at the mid-point of working together on this project in order to ensure that the method of involvement was sustainable and was working for all members of the team [7]. Throughout the lifetime of the RECAP project, eight consumers have contributed to the study, with the current team comprising six experienced consumers.

## Evaluation: a methodology

This evaluation of consumer involvement in the RECAP study took place at the conclusion of the project. Choosing the right evaluation tool to best capture the complexity and impact of the consumer experience in this project was critical to the overall success of the evaluation process. Given this, two comparative scoping reviews of existing consumer involvement evaluation tools were consulted [9, 10]. After considering the recommended tools, the study team decided to use the Australian Clinical Trials Alliance (ACTA) evaluation tool, which forms part of their “Consumer Involvement Toolkit” [11]. This tool was chosen due to its relevance to the Australian context, the design of the RECAP study (as a single-arm trial), its suitability in terms of evaluation length, and the utility of the reflective responses with respect to generating outcomes and learnings for the team. The ACTA evaluation tool was also favoured by our group as it offers a two-pronged evaluation, measuring the process of consumer involvement and the impact of consumer involvement. As the co-design methodology has been important to the development of this team, and as the team plan to continue their work together in the future, it was valuable to be able to evaluate both the process and the impact of consumer involvement.

A member of the study team transferred the ACTA questionnaires (https://involvementtoolkit.clinicaltrialsalliance.org.au/toolkit/evaluating/evaluating-involvement/), into a REDCap survey which was then sent to all members of the study team (researchers, project staff, and consumers). Responses were completed anonymously, and two members of the research team then read and re-read the responses, utilising an inductive thematic methodology to derive major common themes from responses to part one of the evaluation questionnaire (focused on process), and grouping responses under the existing key study domains in part two of the evaluation questionnaire (focused on impact). As agreed by the group, the anonymous responses to the evaluation were then shared in this thematically coded format and discussed at a team meeting, with learnings identified. The suitability of this ACTA evaluation tool for capturing the experience of the team and the impact of consumer involvement was also discussed, with reflections outlined in this paper.

## Evaluation: consumer involvement process

Reflecting on the process of consumer involvement, consumers and researchers were encouraged to think about what happened in the research and how [12]. The ACTA evaluation of consumer involvement form comprises three broad questions for all members of the study team (What do you think is working well? /What do you think are the challenges? /What do you think could be improved? ), and a table of questions with Likert scale responses to be completed by consumers only (Table 1). Conducting an inductive thematic analysis [13] of the responses to these open-ended questions generated key themes of inclusivity, communication, workload for consumers, and remuneration.

### Inclusivity

Several team members highlighted feelings of inclusivity and respect in their reflections on what is working well in the group:

“The building of the relationships of the team. I find this a very inclusive team.” (Respondent 1).

“All are respectful and conscious of building the most supportive dynamic possible for the group.” (Respondent 3).

“Confidence between members to speak freely and be heard respectfully.” (Respondent 9).

“Organisationally and administratively the team has every opportunity to be involved and all are valued by each other.” (Respondent 7).

These responses illustrate the centrality of a dynamic of inclusivity, respect, and appreciation for all members of the team, including how consumers and researchers alike think about a successful consumer-researcher interaction. These values are fundamental to sustainable co-design activity. Further, these reflections demonstrate the importance of considering inclusivity not as an outcome (something achieved), but as a process that must be continually practiced in each interaction and each iteration of consumer involvement in research.

The team also highlighted ideas of inclusivity when reporting on the challenges of consumer involvement. One team member wrote that the team would have benefitted from

“the opportunity to have a broader and more diverse group within the team, eg NESB [non-English speaking background], younger people, so that a more varied voice is heard.” (Respondent 9).

Considering diversity as part of the inclusivity of the group invites an important perspective on the gaps within the lived experience of group members. This has led to fruitful and reflective discussion and, in future projects conducted by this team, focussed efforts will be made to meaningfully and appropriately engage a more diverse range of consumer voices to further the impact of our projects.

### Communication

Clear, respectful, and timely communication is central to sustainable and meaningful bi-directional consumer engagement. Responses in this evaluation focused on feedback as an essential component of this communication, suggesting that consumers value receiving feedback on their input, and also receiving feedback when suggestions cannot be incorporated:

“The active feedback and inclusion of consumer views and if not included a reason as to why not.” (Respondent 1).

Providing feedback to consumers about both the impact of their contribution and the impact of the research project appropriately empowers consumers as members of the study team. Responses in this evaluation highlighted a desire for ongoing feedback to consumers about how the personalised care plan is being received by patients and clinicians, an activity that will extend beyond the conclusion of the project to close the loop with all consumers involved.

### Workload for consumers

Workload for consumers emerged as something that is considered important by both consumers and researchers in the group. Researchers reflected that they were conscious of asking too much of consumers in the project:

“We aim to empower the consumers to say no if we are overburdening them with work but it’s hard to gauge.” (Respondent 2).

“Conscious of not over-burdening the consumers.” (Respondent 3).

Some consumers reflected that sometimes fitting in the work is challenging with their other commitments, while others suggested that they would like to devote more time to the project, with more regular meetings and emails. This range of responses indicate the importance of communicating openly about concerns over workload (too much, or possibly too little where consumers are highly engaged and motivated). It is important to consider the level of commitment and time taken and to actively ensure that the team feels they can contribute to the degree that they would like to and are able to.

### Remuneration

“The more we are asking of people the more it reinforces they should be offered renumeration.” (Respondent 2).

“Another challenge is the lack of funding available to continue remunerating the team.” (Respondent 3).

Remuneration emerged as a particular topic of interest for several members of the study team. Remuneration was considered by all of the research team as essential to supporting the ongoing partnership between consumers and researchers, and is front of mind when planning and budgeting for future projects.

**Table 1:** Consumer reflection on involvement in the RECAP project

Do you feel that…	Not at all	A little	Quite a lot	A lot
you are clear about what your role is (including what you can and cannot change about the project)? (*n* = 6)		1	2	3
you are making a contribution to the research/project? (*n* = 6)		1	3	2
your contribution is valued? (*n* = 6)			1	5
you have enough training/support to undertake your role? (*n* = 6)		1	1	4
you are gaining new skills/knowledge that are useful (for example, you are learning more about the disease condition being researched)? (*n* = 6)		1	3	2
there was enough support from the team leader / chair (*n* = 6)				6

The table above, completed by consumers only, indicates a spread of experience for consumer members of the team. While written comments in this evaluation form were almost exclusively positive, this data identifies areas for growth for our team. Providing improved clarity around consumer roles, offering regular feedback about the consumer contribution to the project, and highlighting and providing more training/skill development opportunities for the team are useful learnings from this evaluation.

## Evaluation: consumer involvement impact

The ACTA evaluation of consumer impact form aims to capture what is changed as a result of the engagement and how big that change is [12].

This evaluation tool is based on research on critical outcomes of research engagement [1], with associated specific measures. As such, the ACTA evaluation tool encourages reflection on consumer impact across seven key study domains: patient-centredness, meaningfulness, ethical design, feasibility, understandability, generalisability, and legitimacy. These domains and relevant quotes from the study team are outlined in Table 2.

**Table 2:** Team reflections on critical outcomes of research engagement

Patient centred Centred around patients’ values, beliefs and experiences. Anticipates participant issues, respects and reflects patient experience. Engages patients in a respectful, culturally appropriate and condition/ disease-sensitive manner	This project has clear outcomes that improve things for patients. (Respondent 1) It has utilised the lived experience of consumers most of whom are/were patients themselves (Respondent 7) The RECAP project is engaging effectively with patients, so it appears the project is well targeted (Respondent 9) There were an appropriate number of consumers involved so an opportunity for a wide range of views (Respondent 10)
Meaningful Research/methods/outcomes are reflective of and relevant/meaningful to the community as well as impactful (as perceived by community and all stakeholders).	*Especially when we had lengthy delays during parts of the project*,* I thought having the patient centred outcomes in mind really helped.* (Respondent 2) *The lived experience of the consumers allowed them to provide an insight of what information is needed by patients* (Respondent 4) *The care plan was very much produced for patients* (Respondent 5)
Ethical design The study optimally protects the rights, safety and well-being of participants	*Ensuring that patients would have the best chance to understand the study and be confident about the storage of their data* (Respondent 3) *The best ways to go about consent…how to approach and conduct the patient interviews* (Respondent 4) *Certainly patient safety and privacy were ongoing considerations by all member of the team– not just the consumers* (Respondent 5) *Definitely in relation to patient privacy and ensuring the informed consent was clear and simple but still covered the appropriate information* (Respondent 10)
Realistic and feasible The study is designed to be patient-centric. The burden on participants of study requirements has been reduced to a minimum.	*Yes*,* this was a key part of things. Having contributions from people with different backgrounds or ways of thinking allowed us to think through some of the practicalities and foreseeable problems.* (Respondent 2) *The consumers provided good insight on when to best receive the care plan (on or after the second appt)* (Respondent 4) *All the team thought outside the box to discuss different ways to approach issues and find the most practical ways to draft the personalised care plan* (Respondent 6) *The consumers were very aware of ensuring minimal burden on patients who have in most scenarios have just received a cancer diagnosis* (Respondent 10)
Understandable Patient/community- facing documents were written in appropriate language and confirmed as readable for the intended audience.	*Documents were considerably better after consumer involvement. There was significant change and evolution in a positive way.* (Respondent 2) *Much time was spent attempting to ensure all information presented was clear*,* understandable and engaging. It appears from the numbers involved in the project that this has been successful* (Respondent 9)
Generalisable Research is appropriately generalisable and the reports of the research make it clear who the study results apply to.	*In general*,* I think researchers and clinicians think in specifics (i.e. drug side effects etc.)*,* and the consumers were good at reminding us to discuss things in a broader nature as well* (Respondent 4) *The co-design process meant that the care plan can be used meaningfully after the study.* (Respondent 5) *The project always looked at the broader patient community i.e. the ways the personalised care plan could/would work to improve the patient journey regardless of their specific cancer or background* (Respondent 6) *Future directions suggest that patients with other types of cancer could benefit from similar care plans* (Respondent 9)
Legitimate The findings are considered legitimate and trusted by patient communities, increasing the likelihood that the results will be translated/adopted due to buy-in.	*Yes*,* inherently a more patient friendly document would be better used.* (Respondent 2) *The team have been consistently reflective if their practice over the course of working together on this project*,* making sure that all decisions are made together*,* as a team* (Respondent 3) *I think the consumer involvement would certainly have influenced ease of use and in that regard adoption might be encouraged. How the plan is introduced to the patient would definitely also have an influence on adoption and this would be done in the clinic* (Respondent 5)

The consideration of the impact of consumer involvement on key study criteria produces comparable and replicable evaluation data. All responses in this evaluation described the positive and productive impact of consumer involvement on the seven criteria, with some offering examples to illustrate this impact with reference to specific tangible changes to the study methodology, materials, and outcome measures.

## Evaluation reflections

Following the completion of the ACTA evaluation forms, consumers and researchers in the team were asked to engage in an informal reflective discussion about their experience of using the evaluation tool. This discussion generated the following key common reflections.

The length of this evaluation tool was generally thought to be acceptable by both consumers and researchers. It was highlighted that this exercise had combined two available ACTA evaluation tools, to capture both the experiences of the consumer involvement process and to measure the impact of consumer involvement on the RECAP study. The average time taken to complete this evaluation, captured as part of the functionality of the REDCap survey, was approximately 26 min. While this was viewed as a manageable length by the team, it is worth noting that the open-ended nature of many of the questions can make the length of the evaluation vary greatly, as demonstrated by the range of response times (which ranged from 11 min through to one hour and eighteen minutes). Selecting an evaluation that is a suitable length to support gathering in-depth and meaningful evaluation data, while at the same time not overburdening respondents, is essential to ensuring that an evaluation process is appropriate and sustainable.

The ACTA evaluation tool was relevant to the patient-centred RECAP study. The team reported that all questions were congruent with their experience and understanding of being involved in the RECAP study. Further, having the seven common criteria against which the impact of consumer involvement in a clinical trial is measured allows reliable data to be produced within research groups/projects (if the evaluation is repeated at multiple points) and across studies (with reflections on these key criteria able to be reported in a standardised way and thus compared).

The style of the questions garnered divergence t within the group. Opinions differed on whether the questions were repetitive or suitably broad to capture a variety of different aspects of consumer involvement. Some felt that the questions seemed too specific, not allowing for narrative reflections on the experience of consumer involvement. Equally, specificity was highlighted by others as a strength of this evaluation tool, prompting recall of activity over the two years being involved in the RECAP project, and allowing respondents to answer in the most considered way. This tension between vagueness and specificity reflects the desire for evaluation tools to be comprehensive, that is to capture the experience and impact of consumer involve in research in a holistic and representative way. This might be best achieved through a balance of broad and specific questions, which both prompt recall and allow space for in-depth reflective writing.

The question of how customised evaluation questions should be to the study they are evaluating was raised. The group discussed the benefit of customising an evaluation tool for a specific study, leading to more targeted questions and responses, and prompting thoughts about specific study related activity. However, the group also reflected that a customised study evaluation would be difficult and time-consuming to construct, may inadvertently shape evaluation results, and will not produce evaluation data that can be compared across studies.

Consumers and researchers also reported that the type of responses the tool aimed to get was unclear, with some answering the questions with a single word response, or short dot points, and others engaging in more reflective writing and providing structured examples for each question. It was felt that clearer instructions, or lined response sections may help to indicate the depth of engagement desired for each question.

Evaluation of consumer input, even in co-designed projects, where consumer input is central to the projects is limited. A recent Australian systematic review [14] reported only 3 out of 51 codesigned studies had any reported evaluation, either with reflective feedback or evaluation questionnaire. None of these projects used the ACTA tool for evaluation, and only one used a formal evaluation tool. The ACTA tool was chosen for our patient centred project; but it lacks generalisability and may not be suitable for all projects. Many frameworks exists and there can be no standard recommendation for a tool for a specific project. This limits the context in which this paper can be viewed and there are limited examples of practical use and evaluation of these tools. Our project is further limited by only having written responses and no follow up for queries to the ACTA too completion. This was intentional to provide anonymity to respondents but limited ability to clarify responses.

Another limitation is that despite our consumer group having eight consumers involved over the lifetime of the project, the group lacked diversity. The consumer group did not include any that identify as Aboriginal and Torres Strait Islander or culturally and linguistically diverse populations. Australia is a multicultural country and not having these groups represented may have limited our findings. Including these groups in future research where available will help give a broader perspective on the acceptability of this evaluation tool.

## Conclusion

Our experience demonstrates that the ACTA evaluation tool is both comprehensive and meaningful in evaluating consumer involvement and impact, at least within the context of our project. The combination of open-ended questions, tick the box responses, and reflections structured around key criteria generates multiple forms of data for evaluation and synthesis. The variety of question styles encourages a comprehensive evaluation, as the tick the box evaluation questions produced more varied responses than the open-ended questions, which tended to generate more positive reflections. The open-ended questions about the consumer involvement process produced authentic reflections on practice, as well as some reflections on areas for improvement and learnings. The criteria-based questions used to measure the impact of consumer involvement on the study produced evaluation data that can be used to describe the impact of consumer involvement, and may be used across multiple studies to build evidence of impact. However, as noted by the team in discussions about the usefulness of the ACTA tool, the questions did produce quite repetitive responses, and the criteria seemed inadequately defined to inspire unique reflections in each category.

The length of the tool (which must be considered along with the burden of undertaking and analysing evaluation data), relevance of questions to the particular activity being evaluated, style of evaluation (including the wording and focus of the questions, and the comprehensiveness of the data captured), and the usefulness of the evaluation data produced are all important considerations when selecting a tool to evaluate consumer involvement in research.

Although our evaluation proved effective in evaluating the RECAP project and suggests applicability to similar consumer projects, it is important to also consider the limitations to using this tool. In particular it’s important to consider the other frameworks that exist in this scope and whether they may be better suited to generalise to an individual project.

Our reflections will inform future evaluation activities conducted by this group, and can provide insight into the evaluation process in general, as well as the perceived strengths and weaknesses of this particular evaluation tool, for other teams looking to evaluate consumer involvement in their research.

## Data Availability

No datasets were generated or analysed during the current study.
